# App-based quantification of crystal phases and amorphous content in ZIF biocomposites†

**DOI:** 10.1039/d2ce00073c

**Published:** 2022-03-04

**Authors:** Michael R. Hafner, Laura Villanova, Francesco Carraro

**Affiliations:** Institute of Physical and Theoretical Chemistry, Graz University of Technology 8010 Graz Austria francesco.carraro@tugraz.at; Faculty of Technical Chemistry, Chemical and Process Engineering, Biotechnology, Graz University of Technology 8010 Graz Austria laura.villanova@tugraz.at

## Abstract

The performance of zeolitic imidazolate frameworks (ZIFs) as protective hosts for proteins in drug delivery or biocatalysis strongly depends on the type of crystalline phase used for the encapsulation of the biomacromolecule (biomacromolecule@ZIF). Therefore, quantifying the different crystal phases and the amount of amorphous content of ZIFs is becoming increasingly important for a better understanding of the structure–property relationship. Typically, crystalline ZIF phases are qualitatively identified from diffraction patterns. However, accurate phase examinations are time-consuming and require specialized expertise. Here, we propose a calibration procedure (internal standard ZrO_2_) for the rapid and quantitative analysis of crystalline and amorphous ZIF phases from diffraction patterns. We integrated the procedure into a user-friendly web application, named ZIF Phase Analysis, which facilitates ZIF-based data analysis. As a result, it is now possible to quantify i) the relative amount of various common crystal phases (sodalite, diamondoid, ZIF-CO_3_-1, ZIF-EC-1, U12 and ZIF-L) in biomacromolecule@ZIF biocomposites based on Zn^2+^ and 2-methylimidazole (HmIM) and ii) the crystalline-to-amorphous ratio. This new analysis tool will advance the research on ZIF biocomposites for drug delivery and biocatalysis.

## Abstract 2

We developed and integrated into a web application (ZIF Phase Analysis) a calibration procedure for the rapid and quantitative analysis of crystalline and amorphous ZIF phases from diffraction patterns in biomacromolecule@ZIF biocomposites.

## Graphical abstract

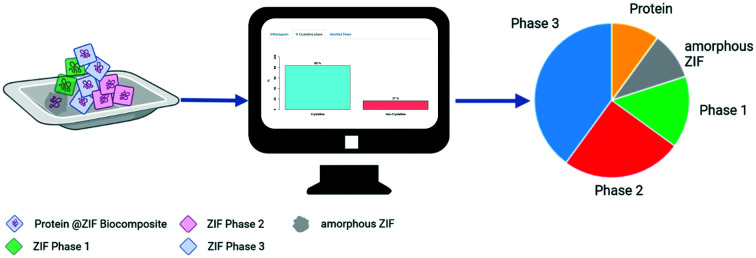

## Introduction

Metal–organic frameworks (MOFs) are a class of coordination polymers that are formed *via* the assembly of inorganic nodes (metal clusters or ions) and organic linkers.^1,2^ Recent progress on composites based on MOFs and biomacromolecules, namely, MOF biocomposites, has been generating significant interest in the field of biotechnology and biomedicine.^3–6^ In particular, zeolitic imidazolate frameworks (ZIFs) prepared from Zn^2+^ and 2-methylimidazole (HmIM) are widely studied because their facile synthesis is compatible with several different classes of biomacromolecules (*e.g.*, proteins, carbohydrates, lipids, nucleic acids, viruses, and cells).^3–5,7–11^ By mixing biomacromolecules to an aqueous solution of Zn^2+^ cations and HmIM, it is possible to find conditions inducing the spontaneous formation of the ZIF material around biomacromolecules (*i.e.*, biomimetic mineralization).^3^ Once a ZIF biocomposite (biomacromolecule@ZIF) is formed, the encapsulated biomacromolecule can be protected against harsh environments,^3,12^ can be stored without the need for refrigeration for long periods^5,9,12^ and can be released in a controlled fashion by exposing the biocomposite to chelating agents (*e.g.* EDTA),^13^ acidic environments (*e.g.*, pH ≤ 6.5)^13,14^ and buffer solutions (*e.g.*, PBS).^13–15^

During the preparation of biomacromolecule@ZIF systems, reaction parameters (metal-to-ligand ratio, concentration, stirring or static conditions) and post-synthetic treatments (washing with water or ethanol) strongly influence the crystal structure of Zn(mIM)_2_-based frameworks.^3,16,17^ The type of crystalline phase strongly influences the physical–chemical properties of the final biocomposite.^16–20^ For example, diamondoid (***dia***) ZIF-8 is a MOF non-porous to N_2_ with a Brunauer–Emmett–Teller (BET) surface area of 40 m^2^ g^−1^,^21^ whereas sodalite (***sod***) ZIF-8 possesses permanent microporosity and has a BET surface area of 1200 m^2^ g^−1^.^21^ Consequently, different ZIF structures react differently towards external stimuli like pH changes (*e.g.*, the release time of Bovine Serum Albumin (BSA) from ***dia*** ZIF-8 is 5 times longer than that from ***sod*** ZIF-8 at pH 5.5).^16^ More peculiar properties were shown by amorphous ZIF biocomposites: in drug delivery, pH changes can induce the instantaneous ZIF degradation and release of the biotherapeutics;^22,23^ in biocatalysis, an enhancement of the substrate diffusion and high enzymatic activities in enzyme@ZIF-8 biocatalysts were measured.^22^ We note that the quantification of the amorphous content is a challenging step that could require the combination of different characterization techniques (*e.g.* X-ray diffraction (XRD), N_2_ physisorption, thermogravimetric analysis, infrared spectroscopy (IR), and X-Ray photoemission spectroscopy).^22^ In general, a careful investigation of the obtained biocomposite typically shows a mixture of phases with a dominant component. As these phases have different functional properties, an inaccurate phase identification results in erroneous assessments of their structure–property–function relationships.^16^ Thus, a tool that enables a rapid and accurate phase assessment in ZIFs will help the progress of biomacromolecule@ZIF composites in biomedicine and biocatalysis.^16,22^

Currently, a straightforward tool for the identification and quantification of both the crystalline phases and the amorphous fraction of ZIF biocomposites is missing. Furthermore, powder X-Ray diffraction (PXRD) analysis is among the most time-consuming steps in the development cycle of novel materials.^24^ To have a time-efficient and quantitative analysis of ZIF biocomposites, we developed a web-based platform (ZIF Phase Analysis) that combines the customizable analytical capacities of the R environment^25^ and the intuitiveness of the user-friendly Shiny interface.^26^ The platform is freely accessible *via* a dedicated website (https://rapps.tugraz.at/apps/porousbiotech/start/) or directly at https://rapps.tugraz.at/apps/porousbiotech/ZIFphaseanalysis/. It includes a novel XRD calibration procedure that we developed to quantify the phases which are commonly found when synthesising ZIF biocomposites from Zn^2+^ and HmIM (*i.e.*, sodalite and diamondoid ZIF-8, ZIF-CO_3_-1, ZIF-EC-1, U12, and ZIF-L,^16,19,27–29^ here referred to as ***sod***, ***dia***, ***ZIF-C***, ***ZIF-EC-1***, ***U12*** and ***ZIF-L***) and quantify the amorphous content *via* the analysis of XRD patterns. This tool will facilitate the data analysis and accelerate the research in the emerging field of ZIF biocomposites, allowing researchers from different fields to precisely correlate the functional properties of the ZIF biocomposites (*e.g.*, drug release profiles and biocatalytic activity) to the crystal phase(s) of the biocomposite.

### Calibration procedure

**Fig. 1:**
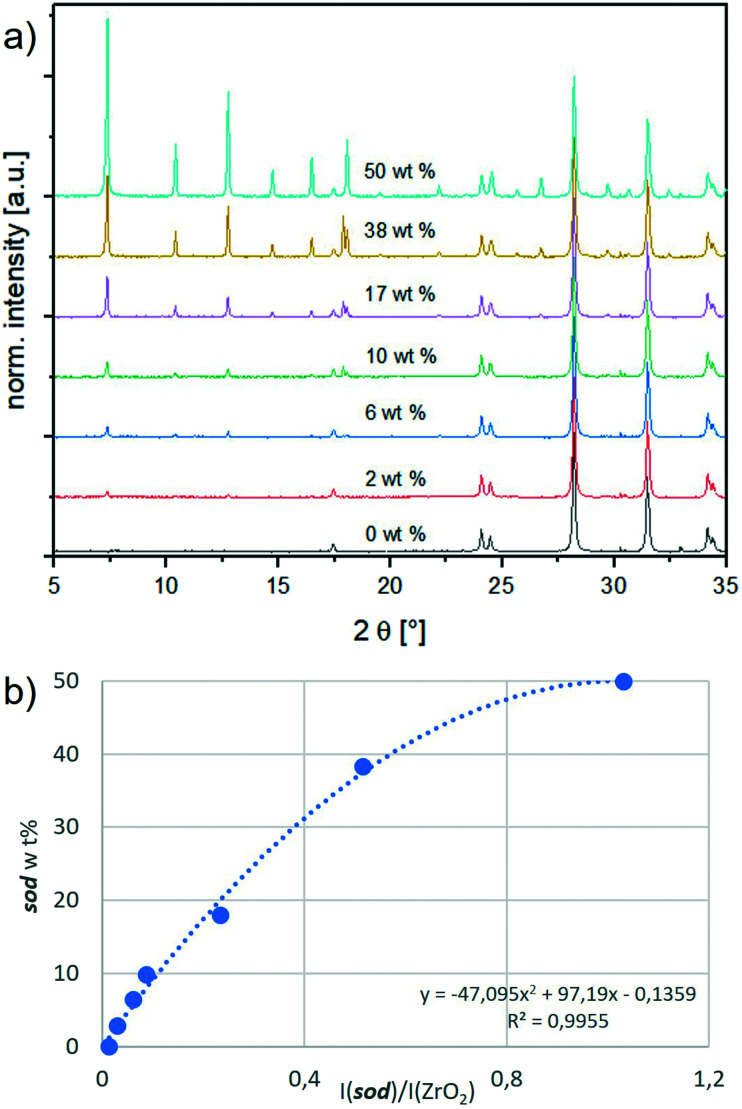
a) Stacked PXRD patterns of ZrO_2_ with an increasing amount of ***sod***, showing the amplified peak at 7.36°; b) calibration curve of sodalite. On the *y*-axis is the weight percentage (wt%) of sodalite in the mixture sodalite/ZrO_2_ and on the *x*-axis is the ratio of the intensities of their respective quantification peaks.

We designed a calibration method with an internal standard (ZrO_2_) for PXRD data analysis. ZrO_2_ was chosen as it is insoluble in water (thus it does not form a MOF with 2-methylimidazole) and its PXRD diffraction peaks do not overlap with ZIF diffraction peaks. Firstly, by optimizing the synthesis conditions, we synthesised each phase – ***sod***, ***dia***, ***ZIF-C***, ***ZIF-EC-1***, ***U12*** and ***ZIF-L*** – in a pure form, excluding the formation of phase mixtures. These reaction conditions can be found in section S2.1.† In Fig. S1,† the PXRD patterns of the different phases are compared to the available simulated patterns to ascertain the type and purity of the obtained phases.^16,17,19,27^ Secondly, increasing amounts of a phase were mixed with the internal standard in different weight fractions (ranging from 2 to 50 wt%; to ensure the reproducibility of the results, we suggest not to use a ZIF wt% lower than 2% when preparing the samples for the calibration curve; see S7†). The detailed procedure is given in section S3.1, and the resulting calibration curves are shown in Fig. S3.†Fig. 1 depicts the calibration curve for ***sod***. The relative ***sod*** weight percent (*i.e.*, ***sod*** wt%) in the ***sod***/ZrO_2_ mixture is plotted in the *y*-axis, whereas the ***sod***/ZrO_2_ integrated intensities ratio (*i.e.*, I(***sod***)/I(ZrO_2_)) is plotted in the *x*-axis. Each integrated intensity is calculated on the quantification peak (QP), namely, the reflection in the diffractogram with the highest intensity. The ***sod*** QP is at 7.36°, and the ZrO_2_ QP is at 28.20° (2*θ*, Cu Kα radiation). The QPs for all phases are listed in Table S1.† Thirdly, for each crystal phase, we determined the ratio between the phase integrated intensity and weight %. We then compared this intensity per weight ratio (phase/ZrO_2_) to a common reference (Al_2_O_3_, corundum) to experimentally derive an instrument-independent constant referred to as the reference intensity ratio (RIR, see Table S1† and section S5).^30–32^

### Crystal phase quantification based on experimental RIRs

In this work, a calibration procedure was used to generate experimentally derived RIRs subsequently exploited for the investigation of ZIF phases (*i.e.*, ***sod***, ***dia***, ***ZIF-C***, ***ZIF-EC-1***, ***U12*** and ***ZIF-L***). Such experimentally derived RIRs were not previously reported in the literature. Instead, the literature reports the ZIF crystal structure from which a RIR can be calculated. However, a calculated RIR does not account for the experimental conditions (*e.g.*, the absence of gas/liquid molecules in the pores) and could drastically differ from an experimentally derived RIR. In our study, the material is dried at atmospheric pressure and room temperature (ESI† for details). Consequently, the calculated RIR differs from the experimentally derived RIR. This difference is more prominent for the porous ***sod*** (the calculated RIR is 10.67,^33^ and the experimental RIR is 4.5) and less prominent for the dense phases (*e.g.*, ***dia***: the calculated RIR is 1.614,^34^ and the experimental RIR is 2.8; ZIF-EC-1: the calculated RIR is 1.403, and the experimental RIR is 2.5). For ***U12***, the experimental RIR is 1.7 (since the crystal structure of ***U12*** is not yet reported, there is no calculated RIR available). In Table S1† the calculated and experimental RIRs of the ZIF phases are listed.

We used the experimentally derived RIRs to enhance the accuracy of a web application that was suitable for the identification of the main crystalline phase by using the calculated or estimated RIRs. Additionally, we revisited the phase selection criteria (section S6†), which now account for additional diffraction peaks and new ZIF phases. These changes resulted in improved identification and quantification of crystalline phases, from traditional (***sod*** ZIF-8) to recently discovered phases (***ZIF-EC-1***).

### Amorphous content quantification (bi-phasic system)

To quantify the amorphous content mixed with a pure ZIF phase (*e.g.*, the amorphous content in a sample that shows only ***sod*** diffraction peaks) we estimated the phase wt% of the pure ZIF phase by using XRD data and its specific calibration curve (ESI† S3.3). Then, we compared this value with the expected phase wt% (*i.e.*, considering the ZIF to be 100% pure and crystalline). If these two values differ, we hypothesise that a part of the material does not contribute to the intensities of the reflection peaks and postulate that the sample is a bi-phasic mixture where a certain proportion of the material is amorphous (*e.g.*, amorphous/pure ***sod***).^32,35^

To verify this hypothesis, we prepared selected protein@ZIF biocomposites that are already reported in the literature.^16^ BSA is used as a model protein, and it is embedded in a ZIF matrix consisting of ***sod***, ***dia***, ***ZIF-C*** or ***U12***. The synthesis protocols are listed in Table S2.†

With FT-IR spectroscopy we verified the formation of the BSA@ZIF composite materials (Fig. S4†).^16^

**Fig. 2:**
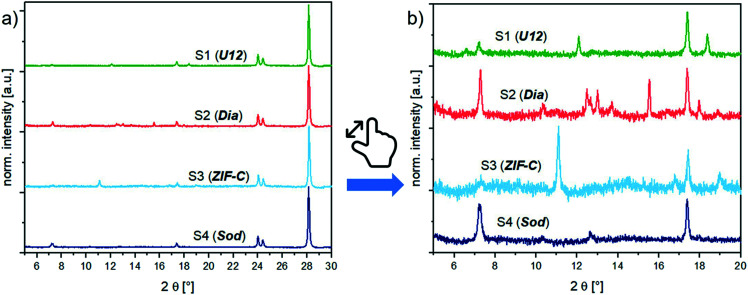
PXRD patterns of the BSA@ZIF biocomposite samples (S1–S4) and their corresponding phases, 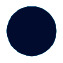: ***sod***, 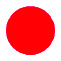: ***dia***, 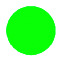: ***U12***, 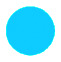: ***ZIF-C***; a) with 50 wt% ZrO_2_ internal standard and b) with 50 wt% ZrO_2_ internal standard, zoomed into the region of the reflection signals of the ZIF phases.

For structural analysis of the BSA@ZIFs composites, we measured the 1 : 1 weight mixtures of BSA@ZIFs composites and the ZrO_2_ internal standard (prepared as in the calibration series, section S3.1†) with PXRD. Fig. 2 shows the diffractograms of the biocomposites. Depending on the reaction conditions (*i.e.*, the metal : ligand : BSA ratio) and the washing procedure (*i.e.*, water or ethanol), different phases like ***sod*** (peaks at 7.36° (110), 10.45° (200) and 12.75° (211)), ***dia*** (peaks at 12.5° (002), 13.05° (011), 13.76° (20–2) and 15.57° (21–1)), ***ZIF-C*** (a peak at 11.05° (110)) and ***U12*** (peaks at 12.13° and 18.43°) can be obtained.^16^

**Fig. 3:**
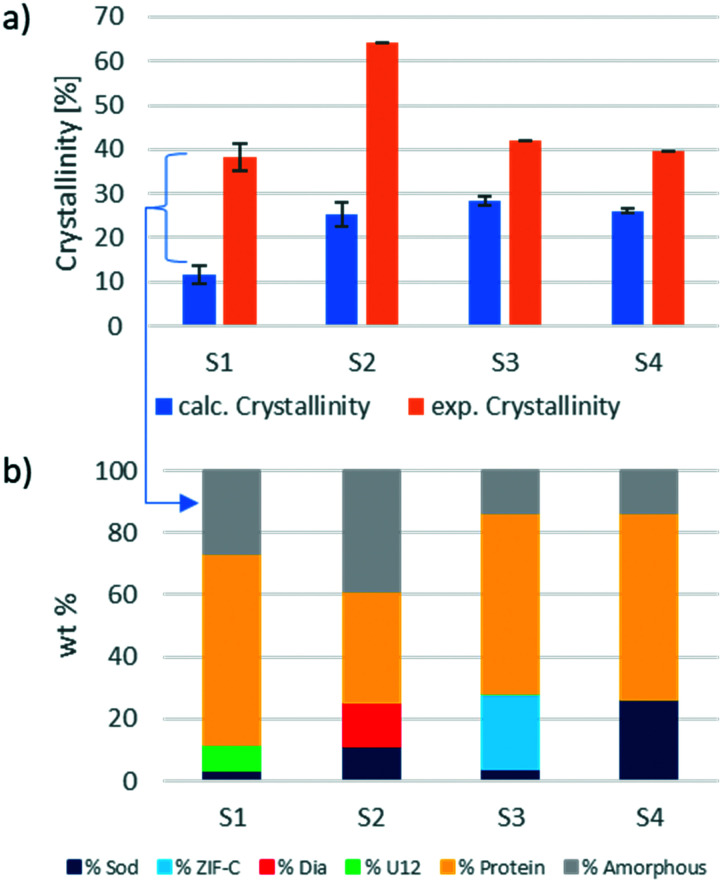
a) Comparison of the calculated crystallinity, obtained through the calibration method with the experimentally found “crystallinity” (amount of ZIF) for the ZIF biocomposite samples (S1–S4). b) Precise phase analysis of the ZIF biocomposite samples (S1–S4) by the calibration method.

However, if the integrated intensity of the QP of the identified ZIF phase is inserted in the calibration curve, the calculated phase wt% is lower than expected. This confirms the hypothesis that only a portion of the weighed material contributes to the diffraction, and thus a certain amount is amorphous. In biocomposites, the formation of an amorphous content seems to be facilitated by the heterogeneous nucleation from the protein.^3,4,36–38^ The amount of encapsulated protein can be calculated *via* the analysis of the protein encapsulation efficiency (EE% determined *via* the Bradford assay; the details are listed in section S4.3†).^16^ The EE% and measured gravimetric yield (Fig. S5 and S6,† respectively) were used to calculate the weight of the encapsulated protein in the investigated sample. By subtracting the weight of the protein from the weight of the total biocomposite material, we determine the weight of the ZIF component for each sample. This theoretical ZIF wt% is then compared to the wt% calculated using the web app. The results of this comparison are shown in Fig. 3. For the analysed samples, the amorphous content ranges from 10% (sample 3) up to 35% (sample 2). Consequently, the amorphous phase has a substantial amount of the ZIF biocomposite, and this should not be overlooked as it can alter the expected properties of the material.^16,22,39,40^

Additionally, this procedure enables the precise evaluation of phase transitions during the processing of the same sample. For example, protein@***ZIF-C*** biocomposites are often synthesized in water; then, when washed with ethanol, a phase transition from ***ZIF-C*** to ***sod*** can be observed.^16^ Here, we examined the conversion of BSA@***ZIF-C*** (sample 3) to BSA@***sod*** (sample 4) *via* ethanol washing. The quantification of the amorphous content in the two samples (15% in both cases) proves that the proportion of the crystalline MOF is maintained during the transition from ***ZIF-C*** to ***sod***. In fact, the ***ZIF-C*** to ***sod*** transition does not involve a sensitive loss of crystalline material (S3: ***ZIF-C*** wt% = 24%, ***sod*** wt% = 4%; S4: ***sod*** wt% = 26%) and supports the hypothesis that ***ZIF-C*** can be fully converted to ***sod***.

### Web app implementation

**Fig. 4:**
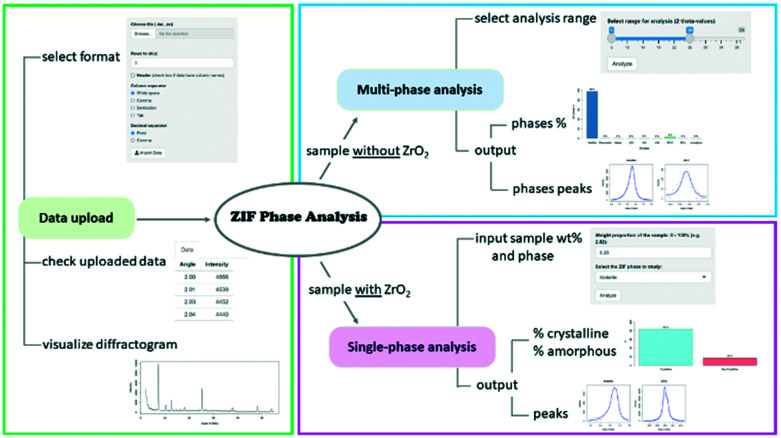
Workflow of the ZIF Phase Analysis app.

The ZIF Phase Analysis app consists of three sections, which are shown in Fig. 4. The data upload section is used to import the diffraction data (see the video in the ESI†). In this section, it is possible to select multiple data file options such as the number of rows to skip and the column/decimal separators. Once the data are uploaded, it is possible to see the first few rows of the data file and the diffractogram.

The multi-phase analysis section is used to analyse samples prepared without the ZrO_2_ internal standard. In this section, it is possible to select the data analysis range by moving the extremes of the slider input to the desired 2*θ* values. We recommend using the 6–28° range as it includes all the reference peaks of the detectable crystal phases. The entire 6–39° range should be used if ZrO_2_ and/or ZnO and/or Zn(OH)_2_ are of interest as well. The analysis returns the identified phases and their relative proportions in the sample, along with the identified quantification peaks.

The single-phase analysis section is used to analyse the samples prepared with the ZrO_2_ internal standard. The user is required to input first the crystal phase to be investigated and then the phase wt% of the studied mixture (*e.g.*, phase wt% = 3%; this implies that ZrO_2_ wt% = 97%). The 2*θ* values are automatically selected based on the crystal phase selected for analysis. The analysis returns the relative proportion of the crystalline and amorphous material in the sample, as well as the identified quantification peaks for both the crystal phase and internal standard. Researchers are encouraged to contact the authors for suggestions on the implementation of additional ZIF biocomposites to the existing web app portfolio.

## Conclusion

We built XRD calibration curves and experimentally determined the reference intensity ratio, RIR, of ZIF phases (***sod***, ***dia***, ***ZIF-C***, ***ZIF-L***, ***U12*** and ***ZIF-EC-1***) that are commonly obtained when preparing ZIF biocomposites. The XRD calibration curves and experimental RIRs were obtained by mixing the different Zn(mIM)_2_-based ZIF phases with an internal standard (ZrO_2_) with different wt% values. The measured experimental RIRs were then used for the crystal phase identification and multiphase analyses. The calibration curves were employed to quantify the amorphous content in the protein@ZIF biocomposite samples. For accurate quantification, an internal standard (ZrO_2_) was used, and the related diffraction patterns were analysed by a customized web application (ZIF Phase Analysis). By examining different protein@ZIF samples with our ZIF Phase Analysis app we estimated an amorphous content of up to 35%. Thus, 1/3 of the entire amount could significantly contribute to the essential functional properties for biocatalysis or drug delivery (enzymatic activity and release profile, respectively). Here we propose a procedure that requires the preparation of protein@ZIF samples and the related standards (controlled mixtures of phases with a ZrO_2_ internal standard). The XRD patterns can then be uploaded in our ZIF Phase Analysis app. The freely available ZIF Phase Analysis app will promptly analyse the data providing an accurate evaluation of the different ZIF phase components, including the amount of the amorphous phase. As protein@ZIF materials is a burgeoning research field approached by scientists with diverse expertise (*e.g.*, biology, enzymology, and biomedicine), this app will especially support researchers with limited knowledge in crystallography. Thus, the here reported procedure combined with the ZIF phase analysis web application will progress research of ZIF biocomposites for multi-disciplinary applications including drug delivery or biocatalysis.^16,22,40^

## Author contributions

M. R. Hafner: design of methodology, experiment implementation, data collection and sorting, discussion of the results and writing of the manuscript; L. Villanova: programming, software development, design and testing of web app, design and launch of website, and revision of the manuscript; F. Carraro: conceptualisation, design of methodology, supervision of the project, discussion of the results and writing of the manuscript.

## Conflicts of interest

There are no conflicts to declare.

## Supplementary Material

CE-024-D2CE00073C-s001

CE-024-D2CE00073C-s002

## References

[cit1] Zhou H.-C., Long J. R., Yaghi O. M. (2012). Chem. Rev..

[cit2] Furukawa H., Cordova K. E., O'Keeffe M., Yaghi O. M. (2013). Science.

[cit3] Liang K., Ricco R., Doherty C. M., Styles M. J., Bell S., Kirby N., Mudie S., Haylock D., Hill A. J., Doonan C. J., Falcaro P. (2015). Nat. Commun..

[cit4] Astria E., Thonhofer M., Ricco R., Liang W., Chemelli A., Tarzia A., Alt K., Hagemeyer C. E., Rattenberger J., Schroettner H., Wrodnigg T., Amenitsch H., Huang D. M., Doonan C. J., Falcaro P. (2019). Mater. Horiz..

[cit5] Herbert F. C., Abeyrathna S. S., Abeyrathna N. S., Wijesundara Y. H., Brohlin O. R., Carraro F., Amenitsch H., Falcaro P., Luzuriaga M. A., Durand-Silva A., Diwakara S. D., Smaldone R. A., Meloni G., Gassensmith J. J. (2021). Nat. Commun..

[cit6] Qiu Q., Chen H., Wang Y., Ying Y. (2019). Coord. Chem. Rev..

[cit7] Poddar A., Conesa J. J., Liang K., Dhakal S., Reineck P., Bryant G., Pereiro E., Ricco R., Amenitsch H., Doonan C., Mulet X., Doherty C. M., Falcaro P., Shukla R. (2019). Small.

[cit8] Zhang Y., Wang F., Ju E., Liu Z., Chen Z., Ren J., Qu X. (2016). Adv. Funct. Mater..

[cit9] Riccò R., Liang W., Li S., Gassensmith J. J., Caruso F., Doonan C., Falcaro P. (2018). ACS Nano.

[cit10] Li S., Dharmarwardana M., Welch R. P., Benjamin C. E., Shamir A. M., Nielsen S. O., Gassensmith J. J. (2018). ACS Appl. Mater. Interfaces.

[cit11] Poddar A., Pyreddy S., Carraro F., Dhakal S., Rassell A., Field M. R., Reddy T. S., Falcaro P., Doherty C. M., Shukla R. (2020). Chem. Commun..

[cit12] Feng Y., Wang H., Zhang S., Zhao Y., Gao J., Zheng Y., Zhao P., Zhang Z., Zaworotko M. J., Cheng P., Ma S., Chen Y. (2019). Adv. Mater..

[cit13] Luzuriaga M. A., Benjamin C. E., Gaertner M. W., Lee H., Herbert F. C., Mallick S., Gassensmith J. J. (2019). Supramol. Chem..

[cit14] Velásquez-Hernández M. D. J., Ricco R., Carraro F., Limpoco F. T., Linares-Moreau M., Leitner E., Wiltsche H., Rattenberger J., Schröttner H., Frühwirt P., Stadler E. M., Gescheidt G., Amenitsch H., Doonan C. J., Falcaro P. (2019). CrystEngComm.

[cit15] Maddigan N. K., Linder-Patton O. M., Falcaro P., Sumby C. J., Bell S. G., Doonan C. J. (2021). ACS Appl. Mater. Interfaces.

[cit16] Carraro F., Velásquez-Hernández M. D. J., Astria E., Liang W., Twight L., Parise C., Ge M., Huang Z., Ricco R., Zou X., Villanova L., Kappe C. O., Doonan C., Falcaro P. (2020). Chem. Sci..

[cit17] Liang W., Ricco R., Maddigan N. K., Dickinson R. P., Xu H., Li Q., Sumby C. J., Bell S. G., Falcaro P., Doonan C. J. (2018). Chem. Mater..

[cit18] Jian M., Liu B., Liu R., Qu J., Wang H., Zhang X. (2015). RSC Adv..

[cit19] Lo Y., Lam C. H., Chang C.-W., Yang A.-C., Kang D.-Y. (2016). RSC Adv..

[cit20] Low Z.-X., Yao J., Liu Q., He M., Wang Z., Suresh A. K., Bellare J., Wang H. (2014). Cryst. Growth Des..

[cit21] Rosen P. F., Calvin J. J., Dickson M. S., Katsenis A. D., Friščić T., Navrotsky A., Ross N. L., Kolesnikov A. I., Woodfield B. F. (2019). J. Chem. Thermodyn..

[cit22] Wu X., Yue H., Zhang Y., Gao X., Li X., Wang L., Cao Y., Hou M., An H., Zhang L., Li S., Ma J., Lin H., Fu Y., Gu H., Lou W., Wei W., Zare R. N., Ge J. (2019). Nat. Commun..

[cit23] Poddar A., Pyreddy S., Carraro F., Dhakal S., Rassell A., Field M. R., Reddy T. S., Falcaro P., Doherty C. M., Shukla R. (2020). Chem. Commun..

[cit24] Oviedo F., Ren Z., Sun S., Settens C., Liu Z., Hartono N. T. P., Ramasamy S., DeCost B. L., Tian S. I. P., Romano G., Gilad Kusne A., Buonassisi T. (2019). npj Comput. Mater..

[cit25] R Core Team , R: A language and environment for statistical computing, R Foundation for Statistical Computing, Vienna, Austria, 2019

[cit26] Chang W. , Cheng J., Allaire J. J., Xie Y. and McPherson J., shiny: Web Application Framework for R, 2020

[cit27] Ge M., Wang Y., Carraro F., Liang W., Roostaeinia M., Siahrostami S., Proserpio D. M., Doonan C., Falcaro P., Zheng H., Zou X., Huang Z. (2021). Angew. Chem..

[cit28] Chen R., Yao J., Gu Q., Smeets S., Baerlocher C., Gu H., Zhu D., Morris W., Yaghi O. M., Wang H. (2013). Chem. Commun..

[cit29] Basnayake S. A., Su J., Zou X., Balkus K. J. (2015). Inorg. Chem..

[cit30] Chung F. H. (2018). J. Appl. Crystallogr..

[cit31] Zhou X., Liu D., Bu H., Deng L., Liu H., Yuan P., Du P., Song H. (2018). Solid Earth Sci..

[cit32] Hubbard C. R., Snyder R. L. (1988). Powder Diffr..

[cit33] Shekhah O., Swaidan R., Belmabkhout Y., du Plessis M., Jacobs T., Barbour L. J., Pinnau I., Eddaoudi M. (2014). Chem. Commun..

[cit34] Shi Q., Chen Z., Song Z., Li J., Dong J. (2011). Angew. Chem., Int. Ed..

[cit35] Hubbard C. R., Evans E. H., Smith D. K. (1976). J. Appl. Crystallogr..

[cit36] Liang K., Carbonell C., Styles M. J., Ricco R., Cui J., Richardson J. J., Maspoch D., Caruso F., Falcaro P. (2015). Adv. Mater..

[cit37] Lian X., Fang Y., Joseph E., Wang Q., Li J., Banerjee S., Lollar C., Wang X., Zhou H.-C. (2017). Chem. Soc. Rev..

[cit38] Ogata A. F., Rakowski A. M., Carpenter B. P., Fishman D. A., Merham J. G., Hurst P. J., Patterson J. P. (2020). J. Am. Chem. Soc..

[cit39] Bennett T. D., Goodwin A. L., Dove M. T., Keen D. A., Tucker M. G., Barney E. R., Soper A. K., Bithell E. G., Tan J.-C., Cheetham A. K. (2010). Phys. Rev. Lett..

[cit40] Liu C., Wang J., Wan J., Cheng Y., Huang R., Zhang C., Hu W., Wei G., Yu C. (2020). Angew. Chem., Int. Ed..

