# Supplementary material for: App-based quantification of crystal phases and amorphous content in ZIF biocomposites
Source: CrystEngComm. 2022 Mar 4;24(41):7266–71. doi: 10.1039/d2ce00073c (PMC9595036; doi:10.1039/d2ce00073c)
Supplement: CE-024-D2CE00073C-s001 [file CE-024-D2CE00073C-s001.pdf]

Electronic Supplementary Material (ESI) for CrystEngComm.
This journal is © The Royal Society of Chemistry 2022

Electronic Supplementary Information

App-based Quantification of Crystal Phases and Amorphous Content in ZIF Biocomposites

Michael R. Hafner, Laura Villanova* and Francesco Carraro*

S1: Materials and Instruments

$Zn(OAc)_2$*2(H_2O) was purchased from EMSURE, Merck. 2-methyl Imidazole (2mIm) was purchased from TCI. $Zn(NO_3)_2$*6(H_2O), ZrO_2 (powder, 5 µm, 99 % trace metal basis, product number: 230693) and Bovine Serum Albumin (BSA, lyophilized powder) were purchased from Sigma Aldrich. Bradford solution was bought from Sigma Aldrich. Silicon wafers (100) were purchased from M.M.R.C. Pty Ltd.

FT-IR measurements were done with an alpha Bruker using transmission mode (resolution 2 cm^{-1}, acquisitions: 128). XRD measurements were done using a Rigaku SmartLab X-Ray Diffractometer (9 kW, Cu anode (λ=1.5406 Å)).

S2: pure ZIF Phases

S2.1: Synthesis of pure ZIF Phases:

Sodalite: 1 ml of an aqueous 160 mM $Zn(OAc)_2$*2(H_2O) Solution was added to 1 ml of an aqueous 2560 mM 2mIm Solution to give a final concentration of $Zn(OAc)_2$*2(H_2O) of 80 mM and of 2mIm of 1280 mM. The reaction was stirred for 24 h. The solid product was separated via centrifugation (13000 rpm for 5 min; centrifuge used: Eppendorf 5425) and the supernatant was discarded. The powder was washed 3 times with 1 ml each de-ionised (DI) water and then air-dried for 48 h at room temperature (23° C).

Diamondoid: 1 ml of an aqueous 80 mM $Zn(OAc)_2$*2(H_2O) Solution was added to 1 ml of an aqueous 320 mM 2mIm Solution to give a final concentration of $Zn(OAc)_2$*2(H_2O) of 40 mM and of 2mIm of 160 mM. The reaction was stirred for 24 h. The solid product was separated via centrifugation (13000 rpm for 5 min; centrifuge used: Eppendorf 5425) and the supernatant was discarded. The powder was washed 3 times with 1 ml each DI water and then air-dried for 48 h at room temperature (23° C).

ZIF-EC-1: 1 ml of an aqueous 160 mM $Zn(OAc)_2$*2(H_2O) Solution was added to 1 ml of an aqueous 320 mM 2mIm Solution to give a final concentration of $Zn(OAc)_2$*2(H_2O) of 80 mM and of 2mIm of 160 mM. The reaction was stirred for 24 h. The solid product was separated via centrifugation (13000 rpm for 5 min; centrifuge used: Eppendorf 5425) and the supernatant was discarded. The powder was washed 3 times with 1 ml each DI water and then air-dried for 48 h at room temperature (23° C).

ZIF-C: 1 ml of an aqueous 30.4 mM $Zn(OAc)_2$*2(H_2O) Solution was added to 1 ml of an aqueous 105.6 mM 2mIm Solution to give a final concentration of $Zn(OAc)_2$*2(H_2O) of 15.2 mM and of 2mIm of 52.8 mM. The reaction was kept at static conditions for 24 h. The solid product was separated via centrifugation (13000 rpm for 5 min; centrifuge used: Eppendorf 5425) and the supernatant was discarded. The powder was washed 3 times with 1 ml each DI water and then air-dried for 48 h at room temperature (23° C).

U12: 1 ml of an aqueous 20 mM $Zn(OAc)_2$*2(H_2O) Solution was added to 1 ml of an aqueous 20 mM 2mIm Solution to give a final concentration of $Zn(OAc)_2$*2(H_2O) of 10 mM and of 2mIm of 10 mM. The reaction was stirred vigorously for 24 h. The solid product was separated via centrifugation (13000 rpm for 5 min; centrifuge used: Eppendorf 5425) and the supernatant was discarded. The powder was washed 3 times with 1 ml each DI water and then air-dried for 48 h at room temperature (23° C).

ZIF-L[1]: 20 ml of an aqueous 0.05 mM $Zn(NO_3)_2*6(H_2O)$ Solution was added to 20 ml of an aqueous 0.4 mM 2mIm Solution. The reaction was stirred for 4 h. The solid product was separated via centrifugation (13000 rpm for 5 min; centrifuge used: Eppendorf 5425) and the supernatant was discarded. The powder was washed 3 times with 20 ml each DI water and then air-dried for 48 h at room temperature (23° C).

S2.2: Characterisation of pure ZIF Phases[1–5]

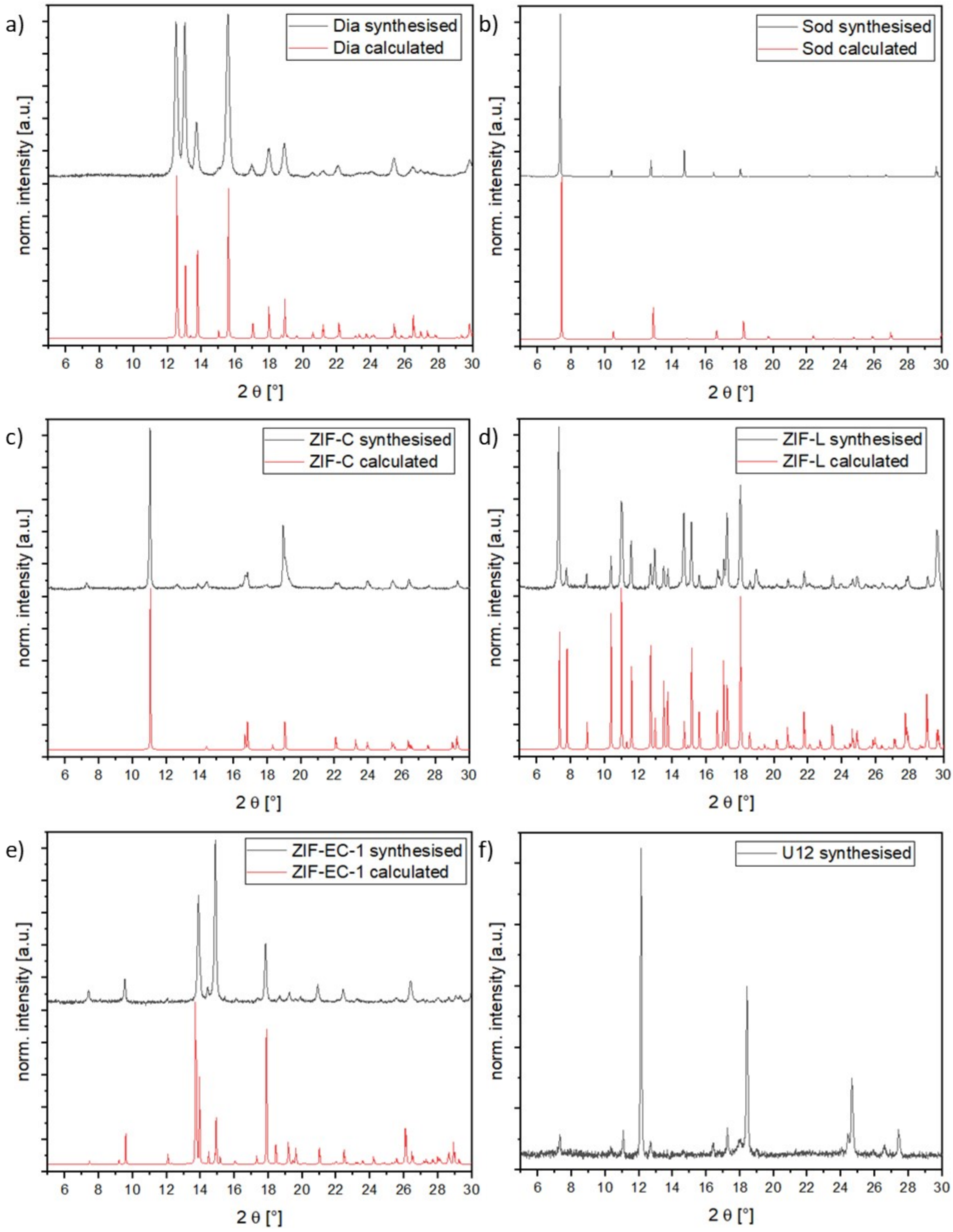

Figure S1: XRD patterns of the pure synthesised ZIF phases and their simulated patterns: a) pattern of synthesised and simulated Diamnondoid, b) pattern of synthesised and simulated Sodalite, c) pattern of synthesised and simulated ZIF-C, d) pattern of synthesised and simulated ZIF-L, e) pattern of synthesised [illegible]

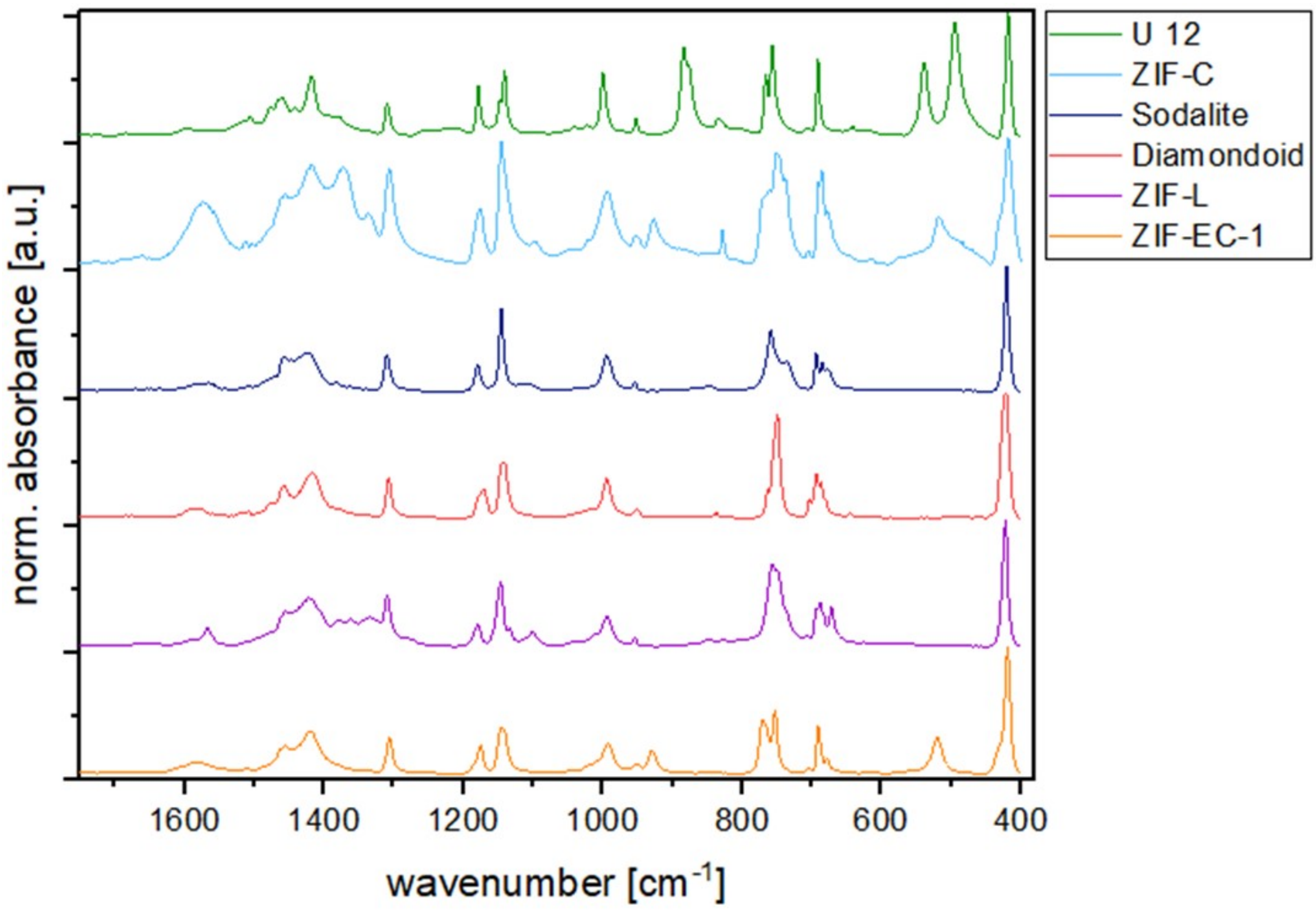

Figure S2: FT-IR spectra of the pure synthesised ZIF phases

S3: Calibration Series

S3.1: Preparation of Calibration Series:

For the calibration of each phase, mixtures with varying content of the pure phase with a constant amount of an internal standard (ZrO_2) were prepared. 5 mg of ZrO_2 were suspended in 150 µl DI water, sonicated for 15 minutes and added to a varying amount of pure ZIF phase (0,1 mg; 0.5 mg; 1 mg; 3 mg; 5 mg). The suspensions were vortexed for 1 minute to ensure a homogeneously mixed suspension. From each suspension mixture 50 µl were drop-cast on a 1.5 cm x 1.5 cm piece of Si and air-dried for 48 h at room temperature (23° C). Triplicates were made from each mixture.

S3.2: Quantification Peaks and Reference Intensity Ratios (RIRs):

Table S1: calculated reference intensity ratios, experimental reference intensity ratios and the quantification peaks of the ZIF phases. The "RIR calc." were calculated using the SmartLab" software (Rigaku) and the .cif structure files.

Phase	RIR calc.[2,6,7]	RIR exp.	Peak (°)
Sodalite	10.67	4.5	7.36
Diamondoid	1.614	2.8	15.57
ZIF-L	1.614	0.9	18.00
U12	n.d.	1.7	12.13
ZIF-C	25.138	2.1	11.05
ZIF-EC-1	1.403	2.5	14.97
ZnO	4.875	5.3	36.30
ZrO_2	4.7	4.7	28.20
$Zn(OH)_2$	1.859	-	24.97

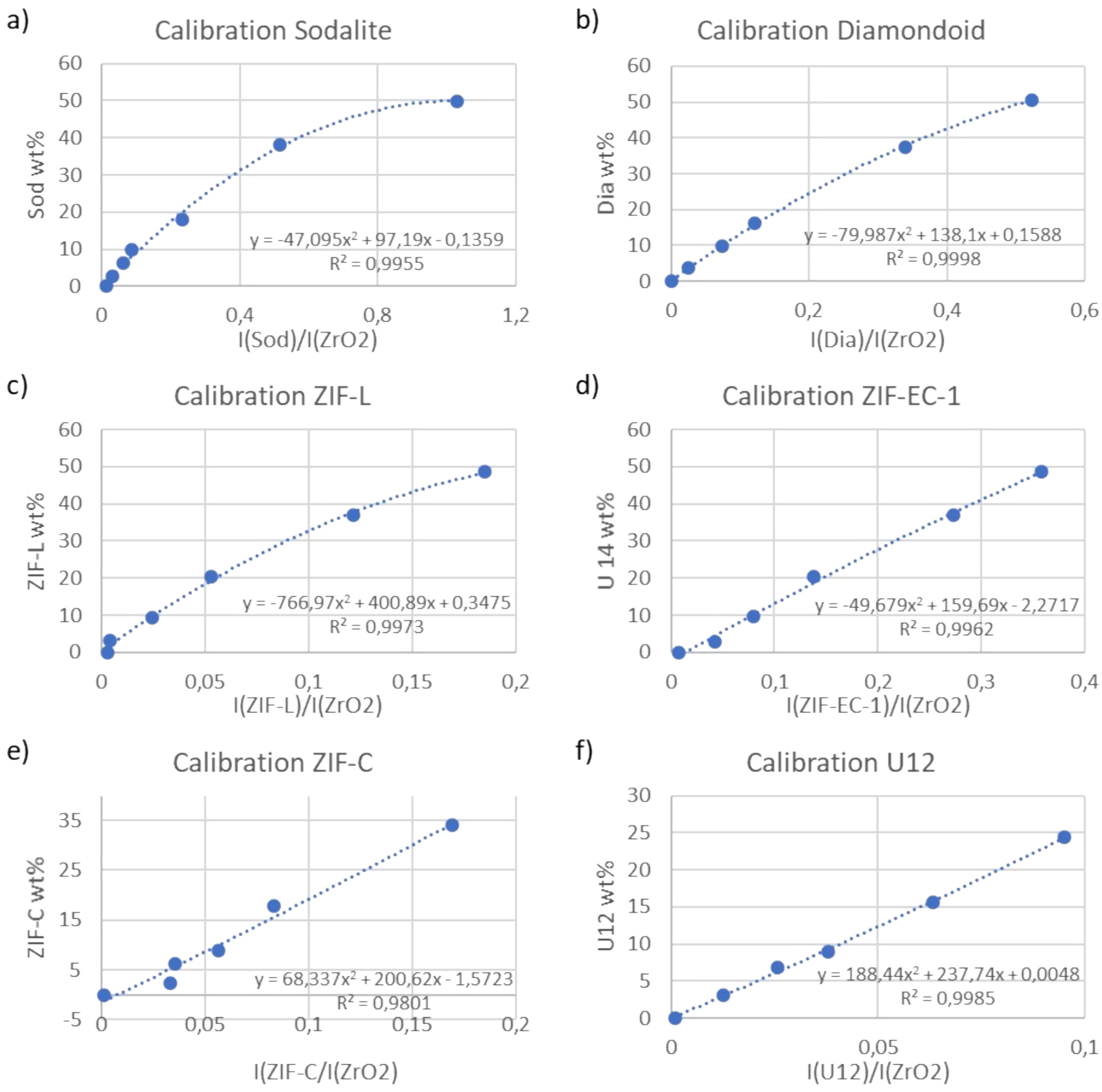

Figure S3: calibration series for a) Sodalite, b) Diamondoid, c) ZIF-L, d) ZIF-EC-1, e) ZIF-C, f) U12

<u>S3.3: Calibration Curves</u>

S4: ZIF Biocomposites

S4.1: Synthesis of ZIF Biocomposites[2]:

4 selected ZIF biocomposite samples (S1-S4) with different phases (U12, Dia, ZIF-C and Sod, respectively) were made according to literature. For this, stock solutions of $Zn(OAc)_2*2(H_2O)$ (160 mM), 2mIm (2560 mM) and BSA (70 mg/ml) were diluted and mixed in varying ratios to yield a final reaction volume of 2 ml. The precise recipe can be found in Table S2. The reaction mixtures were left at static conditions at room temperature (23°C) for 24 h. The solid product was separated via centrifugation (13000 rpm for 5 min; centrifuge used: Eppendorf 5425) and the supernatant was discarded. The powder was washed either 3 times with 1 ml each DI water (*water washing*) or 2 times with 1 ml DI water and subsequently 2 times with 1 ml ethanol (*ethanol washing*). Finally, the powder was air-dried for 48 h at room temperature (23° C).

Table S2: Synthesis protocol for the ZIF biocmoposites[2]

SAMPLE #	Mass Percentage			L/M molar ratio	Mixing volumes (in µL)					
	%	%	%		PRE-MIX L+BSA+water			PRE-MIX M+water		
	M	L	BSA		L	water	BSA	M	water	Washing
S1 (U12)	50	40	10	2.14	40	930	30	310	690	ethanol
S2 (Dia)	30	60	10	5.33	60	910	30	190	810	ethanol
S3 (ZIF-C)	10	80	10	21.30	80	890	30	60	940	water
S4 (Sod)	10	80	10	21.30	80	890	30	60	940	ethanol

S4.2: Characterisation of ZIF Biocomposites

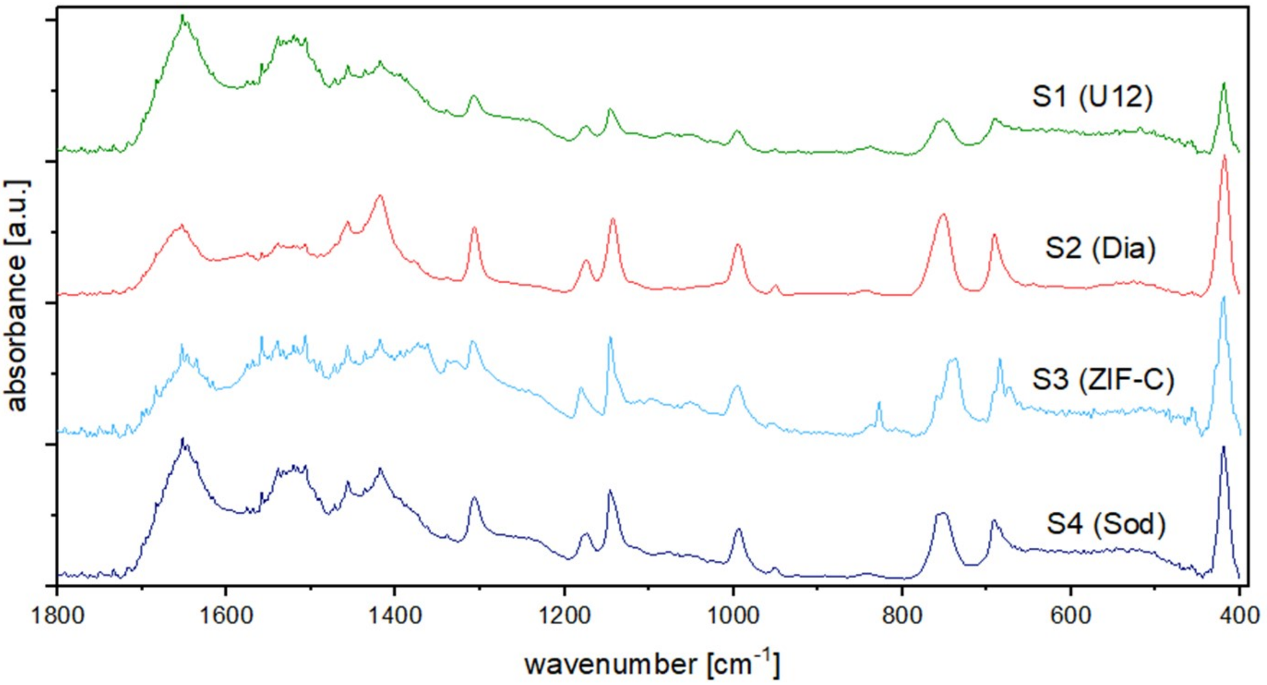

Figure S4: FT-IR spectra of the synthesised ZIF biocomposite Samples (S1-S4) with BSA in the 4 different phases (U12, Diamondoid, ZIF-C and Sodalite)

The bands corresponding to the amid bonds located at 1700-1610 cm^{-1} and 1595-1480 cm^{-1} in BSA are very prominent in the composites.[8,9] Moreover, the peaks at 420 cm^{-1} and 427 cm^{-1}, assigned to the Zn-N stretching in sod (as well as dia, U12, ZIF-EC-1 and ZIF-L) and ZIF-C respectively, are distinct in the spectra.[2,4,10] Depending on the reaction conditions and washing, ZIF-C can be obtained. This can be seen if additional modes are present in the region of 700 to 850 cm^{-1} and 1300 to 1400 cm^{-1}, which are due to stretching modes of the incorporated CO_3^{2-}.[4] Through FT-IR spectroscopy one can therefore get a first impression: if the protein is encapsulated and if ZIF-C is formed.

S4.3: Measurement of Encapsulation Efficiency[2]:

After the synthesis (24h) of a ZIF biocomposite sample, the solid product was separated via centrifugation (13000 rpm for 5 min; centrifuge used: Eppendorf 5425) and the supernatant was recovered. 50 µl of the supernatant were mixed with 1,5 ml of Bradford solution (sample-to-Bradford ratio 1:30). The mixture was slightly shaken by hand and was left at room temperature (23°C) for 15 minutes. Then, the mixture was measured with UV-VIS spectrometry (595 nm).

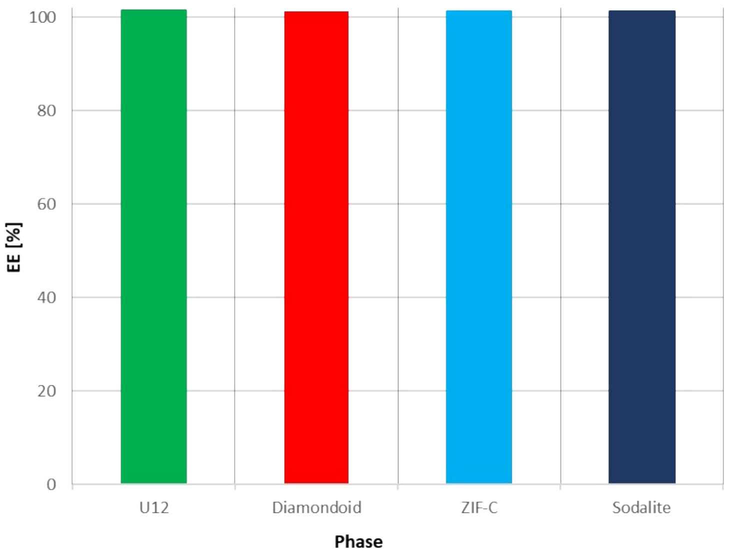

Figure S5: Encapsulation efficiency (EE%) of the ZIF biocomposites with the 4 different phases (U12, Diamondoid, ZIF-C, Sodalite)

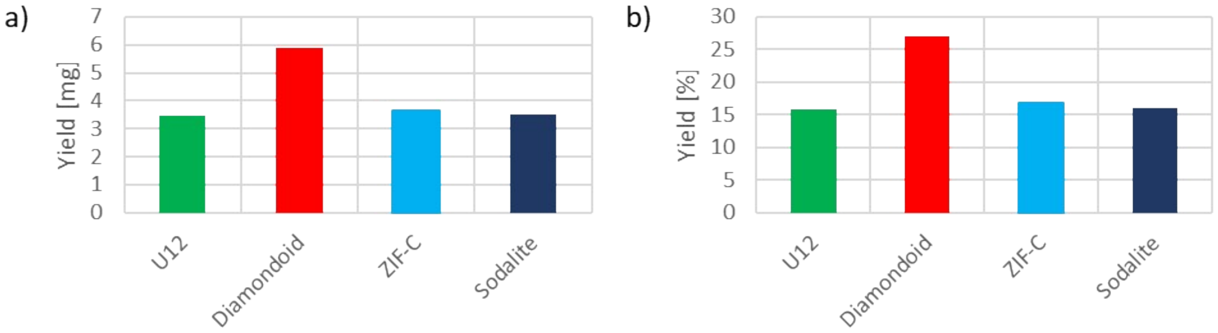

Figure S6: Gravimetric Yield of ZIF biocomposites phases (U12, Diamondoid, ZIF-C, Sodalite) a) total yield given in mg depending on the synthesised phase. b) yield given in percent from the total mass of

S4.4: Yield

S5: Phase and Amorphous Content Quantification

If the reference intensity ratio (RIR) of a material or phase is known, the weight fraction (wt%) of this material or phase (W_i) in a mixture can be determined. This is achieved by comparing the intensity of the most intense peak of the diffraction pattern of the sample (I_i) to the most intense peak of the diffraction pattern of a standard material (I_c), which is usually Al_2O_3 (Corundum). The weight fraction of the standard material (W_c) should be known. This is summarised in the following equation.[11]

$$W_i = \frac{W_c * I_i}{RIR_i * I_c}$$

If Al_2O_3 (Corundum) is not present in the mixture or not available, another reference material (like ZrO_2) can be added as an internal standard (IS). However, the change in the RIR has to be accounted for. A new adapted RIR ($RIR_{i,IS}$) arises, which is a combination of the RIR of the sample and the RIR of the internal standard. One can easily determine the $RIR_{i,IS}$ by mixing a known amount of the IS with a known amount of the sample. The following formula is then applied.

$$RIR_{i,IS} = \frac{I_i / W_i}{I_{IS} / W_{IS}}$$

With this new $RIR_{i,IS}$, there is no longer a dependence on Corundum and it can be replaced in the formula by the IS.

$$W_i = \frac{W_{IS} * I_i}{RIR_{i,IS} * I_{IS}}$$

However, once the $RIR_{i,IS}$ and also the $RIR_{IS,c}$ (the RIR of the IS towards Corundum) are known, the $RIR_{i,c}$ can be calculated from the following formula.

$$RIR_{i,c} = RIR_{i,IS} * RIR_{IS,c}$$

For insights about the crystallinity and amorphous content of the analysed material, the calculated wt% is compared to the actual used wt%.

$$\frac{wt\%_{predicted}}{wt\%_{used}} * 100 = crystallinity\ [\%]$$

The expected crystallinity should be 100 %. However, if this is not met, it means that a certain amount of the material does not contribute to the intensity and is therefore amorphous.[12]

$$amorphous\ content\ [\%] = 100\ \% - crystallinity\ [\%]$$

S6: Phases Selection

For each crystal phase we have identified 3 to 4 reference peaks (Table S3) that are used to uniquely identify the presence of a phase in a diffractogram. Each phase has a quantification peak, which is highlighted in bold in Table S3. It is the most intense peak and typically the first to appear when the phase concentration is low in the sample. The remaining peaks tend to appear as the phase concentration increases in the sample. Additionally, the quantification peak should not overlap with peaks from other phases.

Table S3: *Reference peaks of crystal phases, ZnO and* ZrO_2 *internal standard. The peaks highlighted in bold are the most intense peaks for each phase and are used for the identification and the quantification of the respective phase by the algorithm.*

Phase	Reference peaks (2θ, (*hkl*))			
	**1**	**2**	**3**	**4**
Sodalite	**7.36°, (110)**	12.75°, (211)	16.49°, (310)	18.07°, (22-2)
Diamondoid	12.53°, (002)	13.05°, (011)	13.76°, (20-2)	**15.57°, (21-1)**
U12	**12.13°**	18.43°	24.46°	24.7°
ZIF-EC-1	9.61°, (110)	13.98°, (021)	**14.97°, (200)**	17.94°, (22-1)
ZIF-C	**11.05°, (110)**	12.15°	13.88°	17.87°
ZIF-L	7.33°, (200)	7.77°, (111)	**18.00°, (420)**	-
ZnO	32.10°, (100)	34.4°, (002)	**36.30°, (101)**	-
ZrO2	**28.20°, (11-1)**	31.5°, (111)	34.20°, (002)	-
$Zn(OH)_2$	20.1° (110)	**24.97° (101)**	27.656° (021)	-

The ZIF Phase Analysis app identifies all the peaks in a diffractogram using the diffractometry package[13] and then retain only those peaks that are in the vicinity of the reference peaks. In particular, each identified peak is compared to each reference peak by looking at the difference between the position of the identified peak and the angle of the reference peak. If the absolute difference is 0.10 or smaller, the identified peak is associated to the reference peak. The result is a table similar to Table S3 containing information about the angles of the retained peaks. Based on the retained peaks, the crystal phases present in a sample are determined using the following selection criteria:

1. if only one of the quantification peaks (e.g. Dia peak 15.57°) is identified, then it is assumed that only one crystal phase is present in the sample (e.g. Dia) and the quantification peak is used to quantify the crystal phase;
2. if more than one quantification peak is identified, it is assumed that multiple crystal phases exist in the sample. To select the multiple phases present in a sample we developed an optimized procedure that minimizes the discrepancy between app-based and expert-based phase selection. The optimized procedure selects the Sod, Dia, and/or ZIF-C phase if the corresponding quantification peaks are identified. For the remaining phases, the presence of both the quantification peak and the majority (i.e. 3 out of 4, or 2 out of 3) of the reference peaks is required. Finally, the selected phases are quantified using the corresponding quantification peaks.

It is noted that there exists an overlap between two reference peaks of the Sod phase (7.36° and 18.07°) and the ZIF-L phase (7.33° and 18.00°). To account for this overlap, the ZIF-L phase is considered to be present in the sample only if the peak at 7.77 is identified as well.

S7: Limit of detection

The lowest ZIF wt% experimentally investigated to build the calibration curves was 2% (Fig. S3). Using a 3% wt/wt ZIF-L/ZrO_2, the ZIF-L (the sample with the lowest RIR and therefore the lowest diffraction intensity compared to ZrO_2) diffraction peaks were barely detectable (Fig. S7c,d). Conversely, using the same acquisition parameters (i.e. Cu 9kW source, 4°/min), the quantification peak(s) of the other ZIF phases were clearly identified. We investigated additional wt% (i.e. 1, 0.5, 0.1) of the ZIF phase with the highest RIR (sodalite) to determine its limit of detection in a mixture with ZrO_2. We found that 0.5% wt/wt sod/ZrO_2 is the lowest sod wt% detectable using the same acquisition parameters of the other samples (Fig. S7a,b). However, to ensure the reproducibility of the results for all the phases, we suggest not to use a ZIF wt% lower than 2% when preparing the samples for the calibration curve.

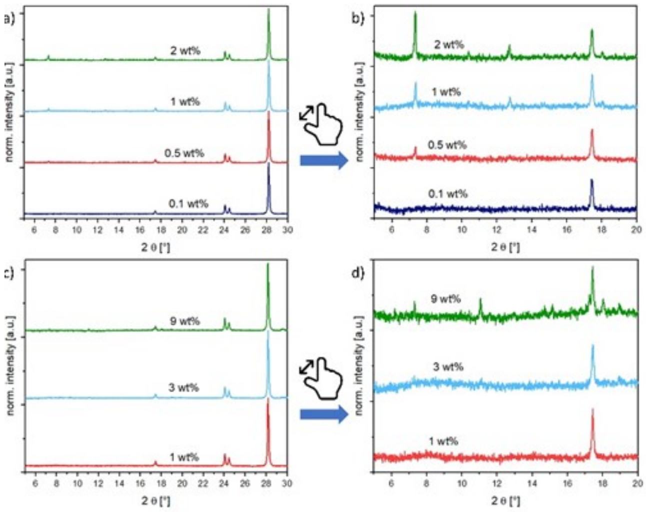

Figure S7: PXRD patterns of the lower ends of the calibration series depicting the limit of detection of Sodalite (a,b) and ZIF-L (c,d) .

References

1 R. Chen, J. Yao, Q. Gu, S. Smeets, C. Baerlocher, H. Gu, D. Zhu, W. Morris, O. M. Yaghi and H. Wang, *Chem. Commun.*, 2013, **49**, 9500.

2 F. Carraro, M. de J. Velásquez-Hernández, E. Astria, W. Liang, L. Twight, C. Parise, M. Ge, Z. Huang, R. Ricco, X. Zou, L. Villanova, C. O. Kappe, C. Doonan and P. Falcaro, *Chem. Sci.*, 2020, **11**, 3397–3404.

3 M. Ge, Y. Wang, F. Carraro, W. Liang, M. Roostaeinia, S. Siahrostami, D. M. Proserpio, C. Doonan, P. Falcaro, H. Zheng, X. Zou and Z. Huang, *Angew. Chem. Int. Ed.*, 2021, **60**, 11391–11397.

4 S. A. Basnayake, J. Su, X. Zou and K. J. Balkus, *Inorg. Chem.*, 2015, **54**, 1816–1821.

5 Y. Lo, C. H. Lam, C.-W. Chang, A.-C. Yang and D.-Y. Kang, *RSC Adv.*, 2016, **6**, 89148–89156.

6 Q. Shi, Z. Chen, Z. Song, J. Li and J. Dong, *Angew. Chem. Int. Ed.*, 2011, **50**, 672–675.

7 O. Shekhah, R. Swaidan, Y. Belmabkhout, M. du Plessis, T. Jacobs, L. J. Barbour, I. Pinnau and M. Eddaoudi, *Chem. Commun.*, 2014, **50**, 2089.

8 K. V. Abrosimova, O. V. Shulenina and S. V. Paston, *J. Phys. Conf. Ser.*, 2016, **769**, 012016.

9 A. Barth, *Biochim. Biophys. Acta BBA - Bioenerg.*, 2007, **1767**, 1073–1101.

10 M. He, J. Yao, Q. Liu, K. Wang, F. Chen and H. Wang, *Microporous Mesoporous Mater.*, 2014, **184**, 55–60.

11 C. R. Hubbard and R. L. Snyder, *Powder Diffr.*, 1988, **3**, 74–77.

12 C. R. Hubbard, E. H. Evans and D. K. Smith, *J. Appl. Crystallogr.*, 1976, **9**, 169–174.

13 P. L. Davies, U. Gather, M. Meise, D. Mergel, T. Mildenberger, T. Bernholt and T. Hofmeister, *diffractometry: Baseline Identification and Peak Decomposition for x-Ray Diffractograms*, 2018, R package version 0.1-10. https://CRAN.R-project.org/package=diffractometry.
